# Blocking of the Larynx by a Caseous Gland

**Published:** 1898-02-26

**Authors:** 


### BLOCKING OF THE LARYNX BY A CASEOUS GLAND.

At the last meeting of the Pathological Society, Dr. Voelcker showed an interesting specimen from a case in which blocking of the larynx had taken place by a caseous gland which had ulcerated its way into the bronchus and been extruded into the trachea, causing death by asphyxia. The specimen was obtained from a child aged five years, who, while apparently in good health, had been left at play, and after twenty minutes had been found dead. The extruded gland had become impacted in the larynx just below the vocal cords, where it has acted as a ball valve, allowing the entry but obstructing the exit of air, and so causing distension of the lungs. Several other examples of the same kind of accident were related. Dr. Cyril Ogle showed a somewhat similar case in which caseous glands had ulcerated through, causing a bulging into the right side of the trachea half an inch above the bifurcation, and practically occluding both bronchi. Dr. Batten said that closure of the bronchial tube by the extrusion of ulcerated caseous glands occurred much more frequently in the right bronchus than the left, and was an important condition to recognise, as the signs closely simulated those of empyema, although there was no cardiac displacement.

